# Adaptive Multivariate Global Testing

**DOI:** 10.1080/01621459.2013.870905

**Published:** 2014-06-13

**Authors:** Giorgos Minas, John A.D. Aston, Nigel Stallard

**Affiliations:** Department of Statistics, University of Warwick, Coventry, CV4 7AL, UK; Division of Health Sciences, Warwick Medical School, University of Warwick, UK

**Keywords:** Adaptive design, Multivariate test, Neuroimaging, Power analysis

## Abstract

We present a methodology for dealing with recent challenges in testing global hypotheses using multivariate observations. The proposed tests target situations, often arising in emerging applications of neuroimaging, where the sample size *n* is relatively small compared with the observations’ dimension *K*. We employ adaptive designs allowing for sequential modifications of the test statistics adapting to accumulated data. The adaptations are optimal in the sense of maximizing the predictive power of the test at each interim analysis while still controlling the Type I error. Optimality is obtained by a general result applicable to typical adaptive design settings. Further, we prove that the potentially high-dimensional design space of the tests can be reduced to a low-dimensional projection space enabling us to perform simpler power analysis studies, including comparisons to alternative tests. We illustrate the substantial improvement in efficiency that the proposed tests can make over standard tests, especially in the case of *n* smaller or slightly larger than *K*. The methods are also studied empirically using both simulated data and data from an EEG study, where the use of prior knowledge substantially increases the power of the test. Supplementary materials for this article are available online.

Supplementary materials for this article are available online. Please go to www.tandfonline.com/r/JASA

## 1. INTRODUCTION

In this work, we develop novel methodology for dealing with recent challenges in testing global hypotheses using multivariate observations. The classical approach for studying the problem, Hotelling's *T*^2^-test (Hotelling 1931), can efficiently detect effects in every direction of the multivariate space when the sample size *n* is sufficiently large. However, in settings where *n* approaches or becomes smaller than the observation dimension *K*, *T*^2^-test becomes respectively inefficient and inapplicable. This cost in efficiency, paid due to the need to search in every direction of the alternative space, seems particularly wasteful (but avoidable), if prior knowledge about the direction of the effect is available. Motivated by the latter settings, often arising in the increasingly important field of neuroimaging, we develop tests which are powerful in studies with *n* ≫ *K*, but can also be efficient in situations where *n* is close to or smaller than *K*.

The proposed tests employ adaptive designs allowing for sequential modifications of the test statistic based on accumulated data. Such adaptive designs have straightforward but not exclusive application in clinical trials. A large literature on the subject (e.g., Bauer and Köhne 1994; Proschan and Hunsberger 1995; Lehmacher and Wassmer 1999; Müller and Schäfer 2001; Brannath, Posch, and Bauer 2002; Liu, Proschan, and Pledger 2002; Brannath, Gutjahr, and Bauer 2012) deals with the derivation of flexible procedures that allow for adaptations of the initial design without inflation of the Type I error rate. Some sequential designs (e.g., Denne and Jennison 2000) also permit design adaptations, but the latter need to be preplanned and independent of the interim test statistics. Adaptive designs are employed for many kinds of adaptations including sample size recalculation (Lehmacher and Wassmer 1999; Mehta and Pocock 2011), treatment or hypothesis selection (Kimani, Stallard, and Hutton 2009), and sample allocation to treatments (Zhu and Hu 2010). Despite the fact that many authors have stressed the potential for test statistic adaptation (e.g., Bauer and Köhne 1994; Bretz et al. 2009), there are only a few papers on the subject (Lang, Auterith, and Bauer 2000; Kieser, Schneider, and Friede 2002). Furthermore, various approaches for adaptive designs in multiple testing are available (see Bretz et al. 2009). These methods can efficiently detect few independently significant outcomes. However, it is well known that standard multiple testing methods (e.g., Bonferroni and Simes tests) become conservative and inefficient in settings, such as the typical neuroimaging studies, where strong dependencies and a large number of outcomes are present (D'Agostino and Russell 2005).

Similarly to the tests developed by O'Brien (1984), Läuter, Glimm, and Kropf (1998), and Minas et al. (2012), the proposed tests are based on linear combinations of the observation vectors. The crucial element in this approach is the weighting vector reducing the observation vectors to the scalar linear combinations. This defines the direction in which we decide to search for effects, and it can substantially affect both Type I and Type II error rate of the tests. O'Brien proposed deriving the weighting vectors under the assumption of uniform mean structure, while Läuter et al. showed that if the weighting vector is derived from the observation sums of products matrix, the Type I error is controlled and high power is attained under certain factorial structures. On the other hand, the tests in Minas et al. (2012) can attain high power levels independently of the mean and covariance structure but a part of the sample is used in a separate pilot study to learn the weighting vector.

In this work, linear combination test statistics, initially constructed using weighting vectors derived from prior information, are sequentially updated based on observed data at subsequent interim analyses in an adaptive design. Early termination of the study (due to early acceptance or rejection of the null hypothesis at an interim analyses) which is often of interest, especially in clinical trials, is also possible within our approach. Our methods provide a formal framework for optimally using prior information in constructing test statistics as has been suggested, but not implemented, in earlier papers (Pocock, Geller, and Tsiatis 1987; Läuter, Glimm, and Kropf 1996; Tang, Gnecco, and Geller 1989a).

While our tests maintain the two prime targets of adaptive designs, namely flexibility and Type I error control (Brannath et al. 2012), we also focus on attaining power optimality. Specifically, we employ the methods proposed by Spiegelhalter, Abrams, and Myles (2002) to derive optimal tests maximizing the predictive power of the test at each interim analysis. The methods of proofs can be useful in deriving optimal adaptive designs in more general settings. As we illustrate in Section 3, the results of Theorem 3.1 could be used to derive optimal designs for regression analysis for example.

The power performance of a multivariate test, lying in a possibly high-dimensional design space, can be hard to illustrate and interpret. Therefore, power analysis of multivariate tests is typically restricted to a limited part of the design space. We tackle this problem by reexpressing the *𝒪*(*K*^2^)-dimensional design space as a lower dimensional easily interpretable space that is still sufficient to determine power. The crucial step here is to identify a measure quantifying the angular distance between the selected weighting vector and the optimal weighting vector and proving its sufficiency in computing power. These results provide wide understanding of the behavior of linear combination tests and allow us to extend earlier work on power analysis of single stage (Pocock, Geller, and Tsiatis 1987; Follmann 1996; Logan and Tamhane 2004) and sequential (Tang, Gnecco, and Geller 1989b; Tang, Geller, and Pocock 1993) linear combination tests, beyond low-dimensional observations or specific mean and covariance structures.

We perform extensive simulation studies to explore and compare the proposed and alternative single stage and sequential procedures throughout the design space. We show that linear combination tests outperform Hotelling's *T*^2^-tests for the latter angular distance being below a certain value which, especially for sample sizes close to *K*, can be rather high. We further show that, in contrast to linear combination tests, such as O'Brien OLS test, with fixed weighting vectors, the adaptive linear combination tests can attain high power levels even in situations where the weighting vector selected at the planning stage is orthogonal to the true optimal (where, of course, a nonadaptive test would have zero power asymptotically). The advantages of the proposed tests are also illustrated through a real example taken from an EEG depression study (Läuter, Glimm, and Kropf 1996).

This article is organized as follows. In Section 2, we formulate the class of linear combination tests while in Section 3 we derive optimal, with respect to power, tests in this class. In Section 4, we present the results allowing us to characterize power based on low-dimensional summaries of the design parameters. In Section 5, we discuss the main results of extensive simulation studies performed using the latter results to explore power and compare the proposed tests with alternative global tests under various conditions, while in Section 6 we apply our procedures to an EEG depression study. Section 7 includes a short summary and discussion of the obtained results. Technical lemmas and proofs are provided in Supplementary Material A, while further illustrations of the simulation studies are provided in Supplementary Material B.

## 2. FORMULATION OF *J*-STAGE LINEAR COMBINATION TESTS

In the following, we formulate *J*-stage linear combination *z* and *t*-tests and define their error rate functions. We assume that the *K*-dimensional observation vectors ***Y**_ij_* = (*Y*_*ij*1_, …, *Y_ijK_*)*^T^* of subjects *i* = 1, 2, …, *n_j_*, participating in stage *j*, *j* = 1, 2, …, *J*, of the study, are independent and identically distributed Gaussian random variables

(2.1)
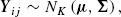


with mean ***μ*** = (*μ*_1_, …, *μ*_*K*_)^*T*^ and covariance matrix the positive definite **Σ** = (*σ_kk′_*)^*K*^_*k,k*′=1_. In medical applications, the mean vector is often interpreted as the treatment effect. We wish to test the global null hypothesis of no treatment effect *H*_0_ : ***μ*** = **0** = (0, 0, …, 0)^*T*^ against the two-sided alternative *H*_1_ : ***μ*** ≠ **0**. Note that the methods which follow equally apply to the two-sample test with common covariance matrix, but we continue with the one-sample presentation to simplify notation.

The observation vectors ***Y**_ij_, **i*** = 1, 2, …, *n_j_*, of the *j*th stage are projected on the nonzero weighting vector ***w**_j_* = (*w*_*j*1_, *w*_*j*2_, …, *w_jK_*)^*T*^ and the projection magnitudes form the linear combinations *L_ij_* = ***w**^T^_j_**Y**_ij_, **i*** = 1, 2, …, *n_j_, j* = 1, 2, …, *J*. The stagewise *z* and *t* statistics for testing *H*_0_ against *H*_1_ using the random sample of linear combinations *L_ij_, i* = 1, …, *n_j_*, when **Σ** is either known or unknown, are respectively

(2.2)
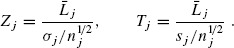


Here, σ_*j*_^2^ is the variance and *L_j_, s_j_*^2^ are the sample mean and sample variance of the linear combination *L_j_*, respectively. Under assumption (2.1), the stagewise *z* and *t* statistics, *Z_j_, T_j_, j* = 1, 2, …, *J* are respectively normally and noncentrally *t* distributed, *Z_j_* ∼ *N*(*θ_j_*, 1) and *T_j_* ∼ *t_ν_j__*(*θ_j_*) with location parameter

(2.3)
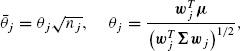


and *ν_j_* = *n_j_* − 1. Under *H*_0_, the *z* and *t* statistics are standard normal and Student's *t* random variables, that is, *Z_j_* ∼ *N*(0, 1) and *T_j_* ∼ *t_ν_j__*. The two-sided stagewise *p* values of the *z* and *t*-tests are, respectively, *p_z_j__* = 2Φ(−|*Z_j_*|) and *p_t_j__* = 2Ψ*_ν_j__*(−|*T_j_*|), where Φ(·) and Ψ(·) are the cumulative distribution functions of the standard normal and Student's *t*-distribution with *ν_j_* degrees of freedom, respectively.

At the *j*th analysis, *j* = 1, 2, …, *J*, performed after the *j*th stage study, a combination function *C*(***p**_j_*) is used to combine the stagewise *p* values, ***p**_j_* = (*p*_1_, …, *p_j_*), of stages 1 to *j* (*p_j_* either *p_z_j__* or *p_t_j__*). Rejection and acceptance critical values *α*_1,*j*_ and *α*_0,*j*_ (0 ≤ *α*_1,*j*_ ≤ *α* < *α*_0,*j*_ ≤ 1, *j* = 1, 2, …, *J*) are used to decide whether to stop the study early and either reject or accept *H*_0_, respectively. Specifically, the *J*-stage sequential design has the following form:

(2.4)
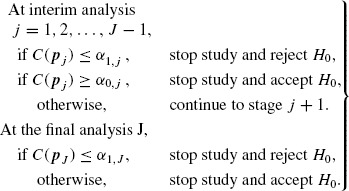


Several combination functions are proposed in the literature. Bauer and Köhne (1994) suggested the use of Fisher's product combination function

(2.5)
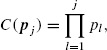


while Lehmacher and Wassmer (1999) suggested the use of the inverse normal combination function. These two combination functions are the most commonly used in the literature (Bretz et al. 2009). The formulation and results which follow use the Fisher's product function in (2.5), but our results equally apply to other combination functions including the inverse normal.

Herein, we will refer to the *J*-stage tests with linear combination stagewise *z* and *t*-test statistics as the *J*-stage *z* and *t*-tests, respectively. The power function, that is, the probability to reject *H*_0_, of the *J*-stage *z* or *t*-test is *β* = Σ^*j*^_*j* = 1_ *β_j_* where, *β*_1_ = Pr(*p*_1_ ≤ *α*_1,1_), the first stage and

(2.6)
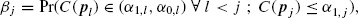


the *j*th stage power functions, *j* = 2, 3, …, *J* (*β, β_j_* either *β_z_, β_z_j__* or *β_t_, β*_*t_j_*_, respectively). The boundaries *α*_1,*j*_, *α*_0,*j*_ are suitably chosen to satisfy the Type I error equation

(2.7)
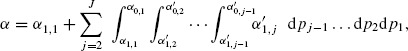


where *α*′_1,*j*_ = *α*_1,*j*_ / *p*_1_*p*_2_ … *p*_*j*−1_, *α*′_0,*j*_ = *α*_0,*j*_ / *p*_1_*p*_2_ … *p*_*j*−1_ the conditional rejection and acceptance boundaries, respectively, of stage *j, j* = 2, 3, …, *J*.

## 3. OPTIMAL *J*-STAGE *z* AND *t*-TESTS

The crucial element for these *J*-stage linear combination *z* and *t*-tests are the stage-wise weighting vectors ***w**_j_*. In this section we develop a methodology for optimally deriving these weighting vectors. The next lemma is the first step for computing the weighting vectors maximizing the power of the *z* and *t*-tests.

Lemma 3.1. Under (2.1), the power of the *J*-stage *z* and *t*-tests in (2.4) with combination function as in (2.5) is nondecreasing in the absolute value of *θ*_*j*_ in (2.3), *j* = 1, 2, …, *J*.

Note that it can be straightforwardly shown that the above result hold for both one-sided stagewise tests and for the inverse normal combination function. The proof of the above lemma is surprisingly complex because for some range of values of *θ*_*j*_ an increase in |*θ*_*j*_| decreases the probability to continue to the next stage and therefore the power of the subsequent stages, *β*^(*j*+1)^ = Σ^*J*^_*l*=*j*+1_ *β_l_*, decreases. In Supplementary Material A, we prove that even for these range of values of |*θ*_*j*_|, the decrease (in absolute value) in *β*^(*j*+1)^ is bounded above by the increase in *β_j_*.

The above result, except for being crucial for deriving Theorem 3.1, can also be useful for more general settings of adaptive designs. For example, Lemma 3.1 proves that if investigators wish to apply an adaptive *z* or *t*-test and are interested in maximizing the power of these procedures, they only need to sequentially maximize the location parameters of the stagewise test statistics separately. For instance, suppose that one is willing to conduct an adaptive design study to explore the relationship between an observation variable *Y* with a set of covariates *X* described by ***Y**_j_* = ***X**_j_**b**_j_* + *e_j_, e_j_* ∼ *N_n_*(0, σ^2^***I**_n_*), *j* = 1, 2, …, *J*, independent. Then, our results prove that to maximize the power of the *J*-stage test with stagewise statistics the classical *z* and *t* statistics, with respect to the experimental design, it is sufficient to maximize *X^T^_j_X_j_, j* = 1, 2, …, *J*, which agrees with the standard practice of deriving optimal designs.

Considering the *J*-stage linear combination *z* and *t*-tests, Lemma 3.1 implies that to maximize the power of these tests with respect to the weighting vectors ***w**_j_*, it is sufficient to maximize the value of *θ_j_, j* = 1, 2, …, *J*. Using this result, we next derive the power-optimal weighting vector.

Theorem 3.1. Under (2.1), the power of the *J*-stage *z* and *t*-tests in (2.4) with combination function as in (2.5) are maximized with respect to the weighting vectors ***w**_j_, j* = 1, 2, …, *J*, if and only if the latter are proportional to

(3.1)
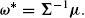

The last result provides the optimal, in terms of power, weighting vector for the *J*-stage linear combination tests ω*. In Section 3.1, we show that ω*, which expresses the multivariate treatment effect standardized with respect to the variance matrix **Σ**, is central in characterizing the power of these tests. However, this optimal vector ω* depends on the unknown parameters ***μ*** and **Σ** and therefore is also unknown. In the next section, we develop a methodology for selecting the weighting vectors ***w**_j_* in practice. We propose using the information for ***μ*** and **Σ**, available at each interim analysis, to optimally select ***w**_j_, j* = 1, 2, …, *J*, where optimality is expressed here in terms of predictive power. The source of this information is the data collected from the stages completed before each interim analysis, but also prior information extracted from previous studies and expert clinical opinion. Predictive power allows the incorporation of this information into our procedures in a natural and plausible way. Note that, as we also explain in the next section, if Equation (2.7) is satisfied, the Type I error of these tests is controlled.

## 3.1 The Proposed z* and t* Tests

Prior information, *ℐ*_0_, is used to inform standard conjugate multivariate priors for the observation mean and covariance matrix. We use the Gaussian–inverse–Wishart prior

(3.2)
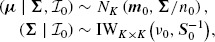


where ***m***_0_ represents a prior estimate of the value of ***μ*** and *n*_0_ corresponds to the number of observations on which this prior estimate is based, while *ν*_0_ and ***S***_0_ respectively represent the degrees of freedom and the (positive definite) scale matrix of the inverse-Wishart prior.

Under this standard Bayesian model (see Gelman et al. 2004), the posterior distribution of ***μ*** and Σ given the information set *ℐ_j_* = {*ℐ*_0_, ***y***_(*j*)_}, consisting of the prior information *ℐ*_0_ and the data collected up to the *j*th interim analysis ***y***_(*j*)_ = [***y***_1_***y***_2_ … ***y**_j_*] is (*μ* | **Σ**,*ℐ_j_*) ∼ *N_K_*(***m**_j_*, **Σ** / *n*_(*j*)_), (**Σ** | *ℐ_j_*) ∼ **IW**_*K*×*K*_(*ν_j_*, ***S***^−1^_j_). Here,

(3.3)
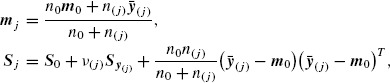


and *ν*_(*j*)_ = *n*_0_ + *n*_(*j*)_ − 1 with *n*_(*j*)_ = *n*_1_ + *n*_2_ + … + *n_j_* and y_(*j*)_ = Σ^*j*^_*l*=1_ Σ^*n_j_*^_*i*=1_ y_*il*_ / *n*_(*j*)_ respectively the sample size and sample mean of ***y***_(*j*)_. Note that, due to the positive definiteness of the prior estimates ***S***_0_, the posterior estimates ***S**_j_* are also positive definite. Positive definiteness of ***S***_0_ is required for our procedures to be applicable.

We wish to use this information to select the weighting vectors ***w**_j_* optimally. Optimality here is expressed in terms of predictive power of the test. Predictive power (Spiegelhalter, Abrams, and Myles 2002) in the present context is derived by averaging the power of the *J*-stage *z* and *t*-tests over the distributions of the model parameters for a given information set. The predictive power for the first stage given the prior information set *ℐ*_0_ is *B*_1_ = Pr(*p*_1_ < *α*_1,1_ | *ℐ*_0_) and for the *j*th stage, *j* = 2, 3, …, *J*, given the information set *ℐ*_*j*−1_ is

(3.4)
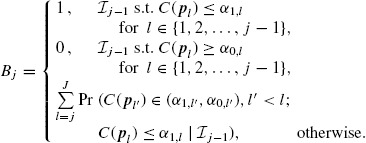


The next result presents the weighting vectors that we suggest to use for the stagewise linear combination *z* and *t*-tests.

Theorem 3.2. Under (2.1) and (3.2), the *j*th stage predictive power, *B_zj_, j* = 1, 2, …, *J*, of the *J*-stage *z*-test in (3.4) is maximized with respect to the weighting vector ***w**_j_* if and only if ***w**_j_* is proportional to

(3.5)
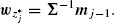

Similarly, as we prove in Supplementary Material A, for *n*_(*j*−1)_ → ∞, the *j*th stage predictive power, *B_tj_, j* = 1, 2, …, *J*, of the *J*-stage *t*-test in (3.4) is maximized with respect to the weighting vector ***w**_j_* if and only if ***w**_j_* is proportional to

(3.6)
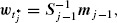


where ***m**_j_, **S**_j_* as in (3.3). The proposed *J*-stage tests, henceforth called (adaptive) z* and t*-tests, proceed as follows: for the *j*th analysis, *j* = 1, 2, …, *J*, (i) obtain w_z*_j__ or w_z*_j__ using (3.5) or (3.6), (ii) set *w_j_* equal to w_z*_j__ or w_z*_j__ and compute the stage *j* statistic *Z_j_* or *T_j_* as in (2.2), (iii) calculate the stage *j p*-value, *p_z_j__* = 2Φ(−|*Z_j_*|) or *p_t_j__* = 2Ψ*_ν_j__*(−|*T_j_*|), (iv) use all the observed *p*-values to perform the combination test in (2.4).

Importantly, the weighting vectors **w**_z*_j__ and **w**_t*_j__, given the prior information and the observed (if any) data ***y***_(*j*−1)_, are fixed before collecting **y**_*j*_ and hence, under the standard conditions described in the following theorem, the Type I error of z* and t*-test, is preserved.

Theorem 3.3. Under (2.1) and for *α*_1,*j*_, *α*_0,*j*_, *j* = 1, 2…, *J* satisfying Equation (2.7), the Type I error of the z* and t*-tests is preserved at the nominal *α* level.

## 4. POWER CHARACTERIZATION (POC)

To study the performance of a test, we primarily need to explore the relationship between its power function and the design parameters. The latter might be, among others, the critical values, the sample size(s), and the model parameters. The critical values and the sample size(s) are scalar and therefore it is straightforward to visualize power even across all their possible values (e.g., using simulations). Their relation to power can then be easily described and understood. In univariate settings, this is also the case for the model parameters. However, in the multivariate setting, model parameters can be high-dimensional and therefore it is not practically feasible to visualize power over the whole design space. Power analysis is then typically restricted to a limited range of different structures of the model parameters. This might be sufficient for power analysis in specific settings, but it has obvious limitations in considering the general behavior of a testing procedure.

In the following, we encounter this problem in the context of linear combination tests and we provide a solution. We first consider the case of *J*-stage linear combination *z* and *t*-tests with fixed weighting vectors which, apart from providing a method for performing simple and efficient power analysis of tests such as the OLS test in O'Brien (1984, see Logan and Tamhane 2004; Pocock, Geller, and Tsiatis 1987; Tang, Geller, and Pocock 1993 for earlier work), also provides the intuition for the results considering the z* and t* tests. Note that in Section 4, the critical values and sample sizes (including the “prior” sample sizes) are assumed to be fixed and described by the design vector ***d*** = (*α*_0,1_, *α*_0,2_, …, *α*_0,*J*_, *α*_1,1_, *α*_1,2_, …, *α*_1,*J*_, *ν*_0_, *n*_0_, *n*_1_, …, *n_J_*).

To provide greater insight to the subsequent results, it is also worth noting the joint distribution of the stagewise linear combination *z* statistics, *Z_j_, j* = 1, 2, …, *J*, here for *J* = 2,


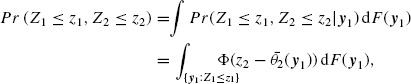


where *F*(***y***_1_) the cdf of the first stage data, ***y***_1_, and *θ*_2_(***y***_1_) the location parameter as in (2.3). The latter parameter is independent of ***y***_1_, that is *θ*_2_(***y***_1_) = *θ*_2_, for the linear combination tests with fixed weighting vector, while for the adaptive z* and t* tests, *θ*_2_(***y***_1_) depends on ***y***_1_ through the weighting vectors in (3.5) or (3.6), respectively. The next section focuses on characterizing further the effect of the weighting vector, through the parameters *θ*_*j*_, on the power function. Note that the power function can be easily derived from the joint distribution of the stagewise statistics by replacing *z_j_* with suitable rejection or acceptance boundaries. In Supplementary Material A, we show that the above expression can be easily generalized to any *J* > 1 and that by replacing Φ(·) with the cdf of the Student's *t*-distribution Ψ(·), we can easily derive the joint distribution of *T_j_, j* = 1, 2, …, *J.*

### 4.1 PoC for the *J*-Stage *z* and *t*-Tests With Fixed Weighting Vectors

To compute the power of the *J*-stage *z* and *t*-tests with fixed weighting vectors ***w**_j_* = ***w***, it is sufficient to know the design vector ***d***, as well as the stagewise location parameters *θ*_*j*_ in (2.3) which in this case are also fixed, that is, *θ*_*j*_ = *θ*. The latter can be reexpressed as

(4.1)
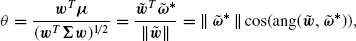


where ang(***w̃***_*j*_, ω̃*) denotes the angle, in measured radians at the origin, between the vectors ***w̃*** and ω̃*. Here, ***w̃*** = **Σ**^1/2^ ***w***, ω̃* = **Σ**^1/2^ ω* = **Σ**^−1/2^ ***μ*** are the standardized selected and optimal weighting vectors. In particular, the latter expresses the standardized multivariate treatment effect, generalizing the univariate (*K* = 1) standardized treatment effect *μ*/σ. Considering the weighting vector selection problem, the first equation in (4.1) implies that a weighting vector that increases the mean and/or decreases the variance of the linear combination gives higher power. The ambiguity in the latter expression becomes clearer by the standardization in the second equation which implies that the weighting vector selection can be expressed as a process of learning the standardized optimal weighting vector ω̃*.

The last equation in (4.1) establishes two scalar measures which are sufficient to determine power. The first is the magnitude of ω̃*, ||ω̃*|| = (***μ***^*T*^ **Σ**^−1^***μ***)^1/2^ = **D**_***μ**, **Σ***_ which is the Mahalanobis distance between the distributions of the observation ***Y**_ij_* under the null and the alternative hypotheses. The Mahalanobis distance is a generalization of the univariate signal-to-noise ratio and can be interpreted as a measure of deviation from the null hypothesis. In medical settings, it is a well-known global measure of the strength of the treatment effect. The second, cos(ang(***w̃***, ω̃*)), is a measure of angular distance between the selected and the optimal weighting vector. It is a measure, in other words, of the distance of our weighting vector selection to the optimal choice. Under this representation, it becomes clear that, for fixed weighting vectors, the location parameter *θ* is equal to a measure (***D_μ,Σ_***) of the strength of the treatment effect scaled down by a measure (cos(ang(***w̃***, ω̃*))) of the distance between the parameters and their prior estimates. The last results are formally stated in the next theorem.

Theorem 4.1. The design vector ***d***, the Mahalanobis distance ***D*_μ,Σ_** = (***μ***^*T*^**Σ**^−1^***μ***)^1/2^ and the angle ang(w̃*, ***ω̃***) between the vectors ω̃* = **Σ^−1/2^*μ*** and ***w̃*** = **Σ**^1/2^***w*** are sufficient to determine the power function *β* of the *J*-stage linear combination *z* and *t*-tests with fixed weighting vectors ***w**_j_* = ***w***.

### 4.2 PoC for the z*-Test

The sequential adaptation of the weighting vector increases the complexity within the relation between the power function and the design parameters. However, following similar methodology as above, analogous results can be derived. For this we use two steps, the first of which involves standardizing the procedure, similarly to (4.1), and the second establishing a rotation invariance property of the power function. The next lemma is a direct consequence of the standardization step summarizing ***μ*, Σ**, and ***m***_0_ to the vectors ω̃* and ***ω̃***_z*_1__.

Lemma 4.1. The design vector ***d***, the standardized optimal weighting vector ω̃* = **Σ**^−1/2^***μ*** and the standardized first-stage weighting vector ***w̃***_z_j*__ in (3.5) are sufficient to determine the power function *β*_z*_.

In the above result, we make use of the fact that the location parameter, θ_z*_j__, of the z*-test can be written as

(4.2)
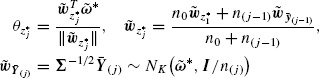


which implies that the adaptive selection of the weighting vectors can be reexpressed as a procedure of adaptive estimation of the vector ω̃*. Under this standardization, we can proceed to the rotation-invariance step which results in the next lemma.

Lemma 4.2. The power, *β*_z*_, of the z*-test is invariant to rotations of the weighting vector ***w̃***_z*_1__ around the optimal weighting vector ω̃*.

The idea behind Lemma 4.2 is that if ***w̃***_z*_1__ is rotated around ω̃*, that is, ***w̃***_z*_1__ is replaced by ***ẇ***_z*_1__ = ***Rw̃***_z*_1__, where ***R*** is a rotation matrix with rotation axis ω̃*, the rejection region of the test is changed. However, the new rejection region is simply a rotation of the initial rejection region. That is, for each point say ***w̃***_*y*_(*j*)__ in the initial rejection region, we can find a unique point, say ***ẇ***_*y*_(*j*)__, in the rotated rejection region such that ***ẇ***_*y*_(*j*)__ = ***Rw̃***_*y*_(*j*)__. Because the symmetrical Gaussian distribution of the observations ***w̃***_*Y*_(*j*)__ ∼ *N_K_*(ω̃*, ***I*** / *n*_(*j*)_) remains unchanged under the rotation, the likelihood of the rejection region, that is, the power of the z*-test, remains the same. The next theorem is direct consequence of Lemmas 4.1 and 4.2.

Theorem 4.2. The design vector ***d***, the Mahalanobis distance ***D***_**μ,Σ**_, and the angle ang(ω̃*, ***w̃***_z*_1__) between the vectors ω̃* and ***w̃***_z*_1__ are sufficient to determine the power function β_z*_.

The above theorem states that the dependence of the power function on the model parameters and their prior estimates is described by simply a scalar measure of the strength of the treatment effect and a scalar measure of distance between the parameters and their prior estimates. It provides a sufficient description of power which is based on easily interpretable summaries and is considerably lower dimensional (importantly not depending on *K*, see Table 1). This allows us to perform power analysis of the adaptive *J*-stage z*-test in a simple way potentially covering the whole design space.

**Table 1.** Model and prior parameters of the z* and t*-tests, respectively, and their dimension

Parameters	Dimension	Parameters	Dimension
***μ*, Σ, *m***_0_	(*K*^2^ + 5*K*)/2	***μ*, Σ, *m***_0_, ***S***_0_	*K*^2^ + 3*K*
ω̃*, ***w̃***_z*_1__	2*K*	ω̃*, ***w̃***_z*_1__, ***D***_1_	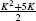
_*D*_*μ*,Σ__, and (ω̃*, ***w̃***_z*_1__)	2	c*, *c*_z*_1__ λ_1_	3*K*

## 4.3 PoC for the t* Test

The need to estimate the unknown **Σ** increases substantially the dimension and the complexity of the design space. The sequential estimation of **Σ**, in addition to ***μ***, to obtain the weighting vectors *w*_t_*_j_, implies that the power analysis needs to account for both estimation procedures. For this, we write the weighting vector ***w̃***_t*_j__, *j* = 1, 2, … *J* in (3.6) as

(4.3)
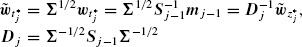


and ***w̃***_z*_j__ the *j*th standardized weighting vector of the z*-test in (4.2). Here the **Σ**-deviation matrix ***D**_j_* is a measure of deviation of the estimate ***S***_*j*−1_ in (3.3) from the parameter **Σ**. The weighting vector ***w̃***_t*_j__ is then written as a product of the inverse of the matrix ***D**_j_*, that accounts for the estimation of **Σ**, and the vector *w̃*_z*_j__ which accounts for the estimation of ***μ***, the latter taking **Σ** as known. We next follow the same steps as in Section 4.2 for deriving the PoC of the t*-test. The standardization step results in the next lemma summarizing ***μ*** and **Σ** and their prior estimates ***m***_0_ and ***S***_0_ to the vectors ω̃* ***w**˜*_z*_j__ and the matrix ***D***_1_ that have clear interpretation.

Lemma 4.3. The design vector ***d***, the matrix ***D***_1_ in (4.3) and the vectors ω̃* and ***w̃***_z*_1__ are sufficient to determine the power function β_t*_.

Here, we use that the location parameter *θ*_t*_j__ and the Σ-deviation matrix ***D**_j_* can be written as

(4.4)
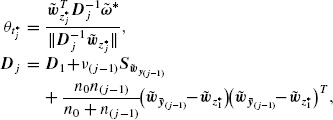


and that ***w̃***_z*_j__ can be written as the weighted average in (4.2). Here, *S****w̃***_*y*_(*j*)__ = Σ^1/2^ *S*_*y*_(*j*)__ Σ^−1/2^ is the covariance matrix of the sample ***w̃***_*y*_*il*__, *i* = 1, 2, …, *n*_*l*_, *l* = 1, 2, …, *j*, where, importantly, ***w̃***_*y*_*il*__ = **Σ**^−1/2^ *Y_il_* ∼ *N_K_*(ω̃*, ***I***).

In a similar fashion to the previous section, we next establish the invariance of the power function under certain rotations of the prior estimates. For this, we define ***V*** = [*v*_1_ *v*_2_ … *v_K_*] to be the matrix with columns the orthonormal eigenvectors of ***D***_1_ and **Λ**_1_ = diag(λ_1_) the diagonal matrix with diagonal λ_1_ = (λ_11_, λ_12_, …, λ_1*K*_)^*T*^ the vector of the corresponding eigenvalues (λ_11_ ≥ λ_21_ ≥ … ≥ λ_1*K*_ > 0). We can then write ***D***_1_ = ***V*** **Λ**_1_***V***^*T*^, ***w̃***_z*_1__ = ***V***_*c*_z*_1___, and ω̃* = ***V***_c_* where

(4.5)
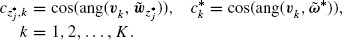


The rotation invariance property of the t*-test is described in the next lemma.

**Lemma 4.4.**

The power function β_t_* is invariant to simultaneous rotations of the vector ***w̃***_z*_1__ and the eigenvectors of the matrix ***D***_1_ around the optimal weighting vector ω̃*.

The proof of Lemma (4.4) is similar to the proof of Lemma (4.2), albeit rather more complex. The next theorem is direct consequence of Lemmas 4.3 and 4.4.

Theorem 4.3. The design vector ***d***, the vector of eigenvalues **λ**_1_ of the matrix ***D***_1_ in (4.3), and the vectors *c*_z*_1__ and c* in (4.5) are sufficient to determine the power function β_t*_.

As we can see in Table 1, the last result reduces the dimension of the design space of the t*-test substantially, allowing us to explore power across the design space. While the design space, due to the covariance matrix estimation, still depends on *K*, it is reduced from order *K*^2^ to order *K.*

Furthermore, this reduction provides an understanding of how the selection of the weighting vector affects power. This becomes clearer if we consider that *θ*_t*_j__ in (4.4) can be written as


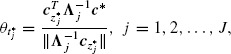


where


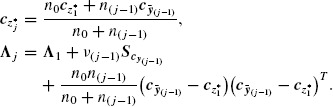


Here, *c*_*y*_(*j*)__ and *S*_*c*_*y*_(*j*)___ are the sample mean and sample covariance matrix of the transformed observation vectors *c*_*y*_***J***__ = [*c*_*y*_1__ *c*_*y*_1__ … *c*_*y*_*j*__] with *c_y_l__, l* = 1, 2, …, *j*, the matrix with columns *c_y_il__* = *V_1_^T^* ***w̃***y*_1_ ∼ *N_K_*(c*, ***I***), *i* = 1, 2, … *n_j_*. The last expressions show that the distance of the prior estimates ***m***_0_, ***S***_0_ to the model parameters ***μ*, Σ** can be expressed by the distances of the vectors c_z_1__* and λ_1_^−1^ = (1/λ_11, …_, 1/λ_1*K*_)^*T*^ to c*, the latter directly reflected to power through θ_t_j_*_ (see the next section for more information).

In the special case of the first stage **Σ**-deviation matrix being proportional to the identity matrix, that is, ***D***_1_ ∝ ***I*** (λ_11_ = λ_12_ = … = λ_1*K*_), as the next result shows, the design space can be reduced further.

Theorem 4.4. For ***D***_1_ = *c*^−1^***I***, the design vector ***d***, the constant *c*, the Mahalanobis distance *D***_μ,Σ_**, and the angle ang(***w̃***_z*_1__, ω̃*) are sufficient to determine the power function β_t_*.

The last theorem proves that, for ***D***_1_ ∝ ***I***, we can use the fact that the prior **Σ**-deviation matrix ***D***_1_ does not change the directions of ***w̃***_z*_j__'s, to show that the relation of β_t*_ to the model parameters and their prior estimates can be described simply by the scalars *D***_μ,Σ_** and ang(***w̃***_z*_1__, ω̃*). In the next section, we use this result and the results of Theorems 4.2 and 4.3 to perform power analysis studies.

## 5. EMPIRICAL STUDIES

To explore properties of the adaptive z* and t*-tests as well as alternative global tests and to perform comparisons, we present empirical studies making use of the results in Theorems 4.2, 4.3, and 4.4.

In addition to z* and t*-tests, we consider linear combination *z* and *t*-tests with fixed weighting vectors, a class that includes the OLS *z* and *t*-test in O'Brien (1984). We also consider the likelihood-ratio χ^2^ and Hotelling's *T*^2^-test with statistics χ^2^ = *n**Y***Σ^−1^*Y* and *T*^2^ = *n*(*n* − *K*)*Y* *S*_*Y*_ / *K*(*n* − 1) that follow the noncentral χ^2^ and *F* distribution with *K* and (*K, n − K*) degrees of freedom, respectively, and noncentrality parameter *D*^2^_*μ*,Σ_. We consider both single stage and sequential *J*-stage designs for all these tests. Finally, the two-step, single-stage linear combination *z*^+^ and *t*^+^ tests proposed in Minas et al. (2012) are also considered. Note that the latter tests can be derived as special cases of the z* and t*-tests for *J* = 2, (α_1,1_, *α*_0,1_) = (0, 1) and *C*(***p***_2_) = *p*_2_.

A range of experiments are performed under different values of the design parameters. The power function of *J*-stage (*J* > 1) tests is not analytically tractable and therefore power is approximated by the rate of rejections in a large number of simulated replications, here *R* = 10,000, of a single experiment. Furthermore, to study the reduction in sample size due to early stopping of the study, we also empirically compute the rate of sample size reduction (RSSR),


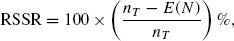


where *n_T_* = *n*_1_ + *n*_2_ + … + *n_J_* the total sample size, *N* the sample size used for a single replication of the study and *E*(*N*) its expected value. Note that single-stage tests have RSSR = 0, in contrast to sequential tests that allow for early stopping and thus have nonzero RSSR.

### 5.1 Simulation Data Examples

We next summarize the main results of a comprehensive study of the power behavior of the above tests in relation to the design parameters (more illustrations are included in Supplementary Material B). First, larger values of *D*_μΣ_ and/or *n_T_* result in higher power values for all tests considered, except the *z* and *t*-tests with fixed weighting vectors ***w̃*** orthogonal to ω̃* for which *β* = *α*. Considering the prior sample size, the results indicate that for *n*_0_ ∊ (0.5*n*_1_, 0.75*n*_1_) the prior estimates become influential, but they do not dominate the accumulated data when selecting the weighting vector while larger values of *n*_0_ enforces z* and t* to have more similar behavior to *z* and *t*-tests with fixed weighting vector. Furthermore, simulation examples confirm that larger values of the acceptance critical values *α*_0,*j*_ increase the power of multistage tests especially for larger potential power gain in subsequent stages, at the expense of less chance of early acceptance. Simulation examples also confirm that larger power is gained if larger rejection critical values *α*_1,*j*_ are allocated to stages with larger potential power gain, while the value of RSSR increases for larger *α*_1,*j*_ in early stages.

**Figure 1.**
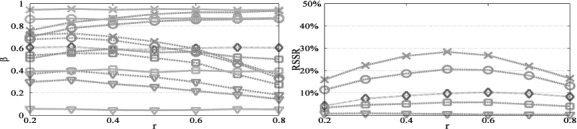
Power (left panel) and RSSR (right panel) versus sample allocation ratio. We plot the sequential χ^2^-test (magenta 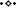) and the z* (green –– line), sequential *z* (cyan –), and *z*^+^ (orange −.) tests with first stage/fixed/first step weighting vector 0 (×), 30° (○), 60° (□) and 90° (∇) angle to the optimal. The remaining design parameters are *J* = 2, *K* = 10, *α* = 0.05, *α*_1,1_ = 0.01, *α*_0,1_ = 1, *n_T_* = 60, *n*_0_ = 0.5*n*_1_, *D*_*μ*,Σ_ = 0.65.

We also consider power behavior related to allocation of sample size to stages (Figure 1). For the sequential *z* and χ^2^-test, the results show that higher power is achieved if sample allocation is analogous to *α*-rate allocation. The z* and t*-tests generally attain higher efficiency for close to balanced allocations. For ***w̃***_z*_1__ close to (far from) the optimal ω̃*, slightly higher power is attained for assigning more sample to early (late) stages. Small to moderate allocation ratios *r* are more appropriate for the *z*^+^ test since no *α* rate is spent in the first stage. Further, as in the χ^2^-test, the z* achieves higher RSSR for *r* = 0.5.

**Figure 2.**
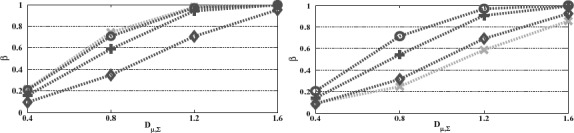
Power of the t*-test versus Mahalanobis distance for various c*, ***c**_z_1__*, **λ**_1_. In the left panel, the vectors c* = ***c***_*z*_1__* ∝ **1** while in the right panel c* = ***e***_1_ = (1, 0, …, 0)^*T*^ and ***c***_*z*_1__* ∝ **1** which, for **λ**_1_ = **1** (green −×− line), give *φ* = ang(c*, **Λ**_1_^−1^ **c**_z_1__*) = ang(c*, λ^−1^_1_) = 0° and 72°, respectively. In both panels, **λ**_1_ 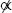 **1** are also chosen to give *φ* = 25° (dark green –o– line), 45° (dark green −+− line) and 65° (dark green –⋄– line). The remaining design parameters are *J* = 2, *K* = 10, *α* = 0.05, *α*_1,1_ = 0.01, *α*_0,1_ = 1, *n_T_* = 20, *r* = 0.5, *n*_0_ = 0.75*n*_1_, *ν*_0_ = *n*_0_ − 1.

Before we proceed to comparisons, it is worth considering the impact of **Σ** being unknown and thus estimated on the performance of the t*-test. First, in the case of ***D***_1_ ∝ ***I*** (λ_1_ ∝ **1** = (1, 1, …, 1)^*T*^), which as we show in Theorem 4.4 is somewhat easier case to consider, the **Σ** estimation variability is substantially reduced and thus we generally expect ***w̃***_t*_j__ to be closer to ***w̃***_t*_j__. On the other hand, if 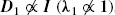, the direction of λ_1_ is more influential on ***w̃***_t*_j__ with the consequence being double-edged (see Figure 2). That is, compared to the situation of λ_1_ ∝ **1**, the distance of ***w̃***_t*_j__'s to optimal can be larger (left panel) but also smaller (right panel) depending on how close the direction of **λ**^−1^_1_ = (1/λ_11_, …, 1/λ_1*K*_)^*T*^ is to the optimal direction c*.

Finally, it is useful to note that throughout our simulations of t*-test, the cos(ang(c*, **Λ**_1_^−1^ ***c***_*z*_*j*__)) is shown to be a robust summary, albeit not sufficient (see Supplementary Material B, Figure 7, Section 2.1), of the distance between the model parameters and their prior estimates. For this reason, but also to reduce complexity, in the comparisons to follow, we focus on the case of **λ**_1_ ∝ **1** (particularly, as we explain later on, in cases resembling the right panel of Figure 2), for various values of the summary cos(ang(c*, **Λ**_1_^−1^ ***c***_*z*_1__*)).

**Figure 3.**
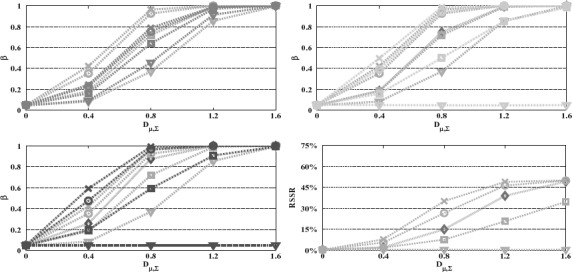
Power and RSSR versus Mahalanobis distance. We plot the z*-test (green ––) with the tests *z*^+^ (orange –.) (up left), sequential *z* (cyan −) and χ^2^ (magenta 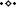) (up right), single stage *z* (blue –) and χ^2^ (red 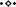) (down left) and sequential χ^2^ (down right). The linear combination z*/*z* / *z*^+^ tests are performed with first stage/fixed/first step weighting vectors having 0 (×), 30° (○), 60° (□), and 90° (∇) angle to the optimal. The remaining design parameters are *J* = 2, *K* = 10, *α* = 0.05, *α*_1,1_ = 0.01, *α*_0,1_ = 1, *n_T_* = 30, *r* = 0.5, *n*_0_ = 0.75*n*_1_, *ν*_0_ = *n*_0_ − 1.

In terms of comparisons, first note that, for fixed design parameters, single-stage tests attain higher power levels than multi-stage tests, nevertheless at the expense of not allowing for early stopping and thus not allowing for sample size reduction (RSSR = 0). Furthermore, it might be useful to emphasize that for fixed design parameters, the power of the linear combination test with weighting vector (either fixed or initial) set equal to the optimal weighting vector ω* attains the maximum power and provides an upper bound to all the other presented procedures, including Hotelling's *T*^2^-test as proved in Minas et al. (2012) (Corollary 1). Compared to the *z*-tests with fixed weighting vectors ***w***, as we can see in Figure 3, the adaptive z* lose some power for ***w̃***(=***w̃***_*z*_1__) close to optimal but gains substantial amounts of power for ***ω̃*** far from optimal, importantly avoiding the problem of *z*-tests having zero power for ***w̃*** orthogonal to optimal. This result emphasizes that, even though the power of the proposed tests remains sensitive to the prior information used to select the weighting vector, they are less sensitive to the initial selection of the weighting vector than the *z* and *t*-tests, where the weighting vector is fixed. The adaptive z*-test also has substantially higher power to *z*^+^ for small angles to the optimal and slightly lower power for large angles. Finally, the power of the single-stage and sequential χ^2^-tests is approximately equal to the power of the z*-test for ***w̃***_z*_1__ having respectively 60° and 45° angle with ω̃*. Note that, as the results in Figure 3 confirm, all the considered tests control the Type I error at the nominal level *α* = 0.05.

**Figure 4.**
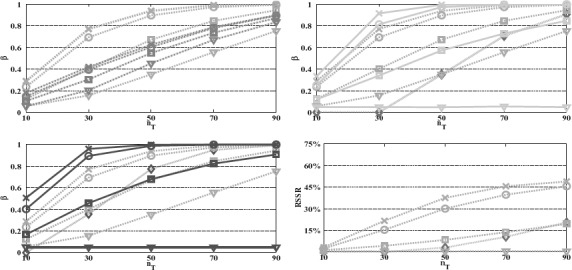
Power and RSSR versus the total sample size *n_T_*. We plot the t*-test (green —.) with the tests, *t*^+^ (orange −.) (up left), sequential *t* (cyan –) and *T*^2^ (magenta 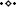) (up right), single stage *t* (blue −) and *T*^2^ (red 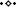) (down left) and sequential *T*^2^ (down right). The linear combination t*/*t* / *t*^+^ tests are performed with first stage/fixed/first step weighting vectors having 0 (×), 30° (○), 60° (□), and 90° (∇) angle to the optimal. The remaining design parameters are *K* = 15, *J* = 2, *α* = 0.05, *α*_1,1_ = 0.01, *α*_0,1_ = 1, *r* = 0.5, *n*_0_ = 6, *ν*_0_ = *n*_0_ − 1, *D*_*μ*,Σ_ = 0.7.

In the case of **Σ** unknown, we consider comparisons for the case of ***D***_1_ = ***I*** which, using the results of Theorem 4.4, they can be performed in a similar way to the case of known **Σ**. For the simulations in Figure 4, the case of ***D***_1_ = ***I*** can be thought of as representative of λ_1_^−1^ fairly distant to c* (right panel of Figure 2), since we take c* = ***e***_1_ resulting in cos(ang(c*, λ^−1^_1_)) = √*K/K* (≅0.26, angle 75°, for *K* = 15). As we would expect, the power of all tests is lower than their counterparts for **Σ** known (same design parameters), but the patterns of power difference across tests remain the same except from Hotelling's *T*^2^ which in contrast to χ^2^-test is highly dependent on the sample size.

As Figure 4 illustrates, for *n_T_* ≤ *K* or *n_T_* slightly larger than *K* (here, *n_T_* = 10–30 for *K* = 15), *T*^2^ is respectively inapplicable or very inefficient with power levels lower than the power of t* even for angles close to orthogonal. As sample size becomes considerably bigger than *K* (*n_T_* > 50), the power of *T*^2^-test increases sharply to yield power levels analogous to the χ^2^-test. For instance, for the design parameters in Figure 4, the single stage and sequential *T*^2^-tests, likewise to the χ^2^-test, have power close to the power of the t* for angle 60° and 45°, respectively, for large sample sizes.

## 6. APPLICATION TO AN EEG STUDY

We consider applications to an electroencephalogram (EEG) study, the results of which are provided in Läuter, Glimm, and Kropf (1996). As Läuter et al. described, the data are collected from *n_T_* = 19 depressive patients at the beginning and at the end of a six week therapy. For demonstration, *K* = 9 variables are used which represent the changes of the absolute theta power in channels 3–8, 17–19 of EEG during the therapy of each patient. In Table 2, we present the means, standard deviations, and correlation matrix of the data. Note that although an increase is indicated in all channels, none of them (min*_k_* *p_k_* = 0.04) fall below the Bonferroni corrected threshold *α* / *K* ≅ 0.0056 at the *α* = 5% significance level. Hotelling's *T*^2^-test also fails to reject *H*_0_ (*p*_*T*^2^_ = 0.261). On the contrary, the SS and PC *t*-tests proposed by Läuter et al. reject *H*_0_ at the 5% significance level (*p*_SS_ = 0.0489, *p*_PC_ = 0.0487).

**Table 2.** Means, standard deviations, correlations, and their prior estimates for the EEG depression study presented in Läuter, Glimm, and Kropf (1996)

ch.	3	4	5	6	7	8	17	18	19
*y_k_*	0.8710	1.5890	1.0370	1.1460	0.8510	0.8530	1.4220	0.7510	0.9950
*m*_0,*k*_	0.5	3.50	1	2	2	2	2	2	2
*s_y_k__*	2.9494	3.5121	2.3637	2.2490	2.2760	2.0706	3.2624	2.6382	2.3593
*s*_0,*k*_	1.5	2.5	1	2	2	2	2	2	2
*R*_0_\*R_y_*	1	0.9262	0.8115	0.7959	0.5786	0.4902	0.9323	0.4896	0.5312
4	0.8	1	0.6270	0.7835	0.3357	0.4450	0.9313	0.2778	0.4892
5	0.8	0.7	1	0.7882	0.8492	0.7173	0.7347	0.7145	0.7611
6	0.7	0.8	0.7	1	0.6020	0.7924	0.8180	0.6334	0.7783
7	0.5	0.4	0.7	0.55	1	0.6155	0.4639	0.6833	0.5992
8	0.4	0.5	0.55	0.7	0.6	1	0.5177	0.5983	0.7833
17	0.9	0.9	0.75	0.75	0.45	0.45	1	0.4048	0.5711
18	0.45	0.45	0.65	0.65	0.7	0.7	0.5	1	0.4445
19	0.75	0.75	0.8	0.8	0.65	0.65	0.8	0.7	1

We perform power analysis by setting the design parameters as in the above study, that is, *n_T_* = 19, *K* = 9, ***μ*** = *y*, **Σ** = ***S**_y_, α* = 0.05. For these design parameters, the power of Hotelling's *T*^2^ is *β_T^2^_ ≅ 0.68 (*D*_μ,Σ_ = 1.15). This is larger than the power of the SS and PC tests which are respectively *β*_*t*_SS__ ≅ 0.52, *β*_*t*_PC__ 0.51 (the contrasting results of the tests performed using these data are because of the different shape of the *t* and *F* distributions). The latter power values are very close to the power of the OLS *t*-test in O'Brien (1984), *β*_*t*_OLS__ ≅ 0.52, which uses the uniform weighting vector ***w***_OLS_ ∝ **1**. This gives angle ang(*w̃*_OLS_*, ***ω̃***) ≅ 71°. Taking into account that the single-stage *t*-test for a weighting vector equal to the optimal has power *β_t_* ≅ 1, we can easily see that there is considerable scope for improvement.

Since the study was performed, there has been considerable research into EEG studies on depressive patients. There is now literature (see, e.g., Davidson et al. 2002) indicating that left-frontal hypoactivation and right-frontal hyperactivation are present in such subjects. This would indicate that a nonuniform prior over these frontal regions should be used. Using prior information based on such evidence, the adaptive t*-test can attain high power levels. For example, the prior estimates given in Table 2 are in agreement with the evidence in the literature and further, the prior correlation structure is set to be roughly coherent to the distances between the channels, that is, larger distances have smaller correlations, with larger correlations set at the highly active frontal regions (in accordance with the literature).

This prior estimate gives ang(*w̃*_t*_1__) = 37.27° which is much smaller than the angle under the uniform weighting vector. For a two-stage design (*J* = 2), with balanced sample allocation, *n*_1_ = 10, *n*_2_ = 9, and *α* allocation *α*_1,1_ = 0.01, *a*_2_ = 0.0087, no early acceptance allowed, *α*_0,1_ = 1, prior sample size *n*_0_ = 7 = 0.7*n*_1_, *ν*_0_ = 6 (see previous section) and the remaining design parameters as the original study, the t*-test has power β_t*_ = 0.84 with RSSR = 22.3% (*E*(*N*) ≅ 15). Substantial power improvement is also obtained over the *t*^+^ which, for *n*_0_ = 6, *n*_1_ = 13, *n*_2_ = 6 (*r* = 0.3) and the remaining design parameters as above, has power *β*_*t*^+^_ ≅ 0.64.

## 7. DISCUSSION

The methods developed in this work demonstrate that linear combination tests provide a substantial alternative to the classical Hotelling's *T*^2^ global test, especially in the setting, commonly encountered in recent important applications of clinical neuroscience, of the available sample size *n* being small compared to the observation dimension *K*. It is also shown that adaptive linear combination tests provide power robustness across the set of alternative hypotheses since they can correct initial selections of the weighting vector which are far from the optimal selection. The adaptive *J*-stage z* and t*-tests achieve high power levels for large *n*, independently of the initial selection of weighting vector, but most importantly they can achieve high-power performance even if *n* is limited.

The proposed tests achieve optimality in the sense of maximizing the predictive power of the test at each interim analysis. Predictive power has been used for sample size calculation (O'Hagan and Stevens 2001), treatment selection (Kimani, Stallard, and Hutton 2009) and to select the component-wise significance levels in multiple testing (Westfall, Krishen, and Young 1998). It is a useful tool for incorporating prior information into the design of a study, particularly as such studies can often be viewed as a decision-making process. The application in Section 6 provides an example of a setting in which prior information is available and can substantially improve the performance of existing tests.

Optimality is attained in our methods without undermining the two main targets of adaptive designs: flexibility and test specificity. This allows for future developments of the proposed test to consider further optimal design adaptations. The use of other adaptive designs techniques, such as sample size reassessment, within our methodology can improve further the performance of the proposed tests.

The power characterization in Section 4 provides a tool for understanding and alleviating to some extent the complexities of multivariate tests especially those based on response dimension reductions. The possibly high-dimensional model parameters and their prior estimates are reduced to low-dimensional summaries which are still sufficient to compute power. Importantly, these summaries have interpretations directly related to the strength of the treatment effect and the effect of the dimension reduction on power. They provide a method for performing simple power analysis, but also understanding the behavior of linear combination tests.

The methods used to derive the power characterization are also interesting in their own right. They can be generally described by two steps: standardization and rotation invariance. The first standardization step is a prevalent technique for reexpressing statistical models in the standard deviation unit and eliminating correlations. Here, it allows us to reexpress the weighting vector selection, which involves estimating the unknown model parameters, as a procedure of learning a single vector, that is, the optimal weighting vector. The second step of establishing a rotation invariance property for the power function allows us to identify the measure quantifying the angular distance between the selected and the optimal weighting vector, reducing further the design space. The question whether these results can be derived under more relaxed modeling assumptions is an area of ongoing research.

## SUPPLEMENTARY MATERIALS

Additional supplementary material is provided in the following documents:

**Supplement A: Technical results** Technical details, lemmas, and proofs.

**Supplement B: Extended simulation examples** Examples from the extensive simulation studies performed to study the power of the considered tests.
